# What is allosteric regulation? Exploring the exceptions that prove the rule!

**DOI:** 10.1016/j.jbc.2024.105672

**Published:** 2024-01-23

**Authors:** Martin McCullagh, Tonya N. Zeczycki, Chathuri S. Kariyawasam, Clarissa L. Durie, Konstantine Halkidis, Nicholas C. Fitzkee, Jo M. Holt, Aron W. Fenton

**Affiliations:** 1 Department of Chemistry, Oklahoma State University, Stillwater, Oklahoma, USA; 2 Department of Biochemistry and Molecular Biology, Brody School of Medicine at East Carolina University, Greenville, North Carolina, USA; 3 Department of Chemistry, Mississippi State University, Mississippi State, Mississippi, USA; 4 Department of Biochemistry, University of Missouri, Columbia, Missouri, USA; 5 Department of Hematologic Malignancies and Cellular Therapeutics, The University of Kansas Medical Center, Kansas City, Kansas, USA; 6 Department of Biochemistry and Molecular Biology, The University of Kansas Medical Center, Kansas City, Kansas, USA

**Keywords:** allosteric regulation, DNA–protein interaction, antibody, molecular modeling, structure–function

## Abstract

“Allosteric” was first introduced to mean the other site (*i.e*., a site distinct from the active or orthosteric site), an adjective for “regulation” to imply a regulatory outcome resulting from ligand binding at another site. That original idea outlines a system with two ligand-binding events at two distinct locations on a macromolecule (originally a protein system), which defines a four-state energy cycle. An allosteric energy cycle provides a quantifiable allosteric coupling constant and focuses our attention on the unique properties of the four equilibrated protein complexes that constitute the energy cycle. Because many observed phenomena have been referenced as “allosteric regulation” in the literature, the goal of this work is to use literature examples to explore which systems are and are not consistent with the two-ligand thermodynamic energy cycle–based definition of allosteric regulation. We emphasize the need for consistent language so comparisons can be made among the ever-increasing number of allosteric systems. Building on the mutually exclusive natures of an energy cycle definition of allosteric regulation *versus* classic two-state models, we conclude our discussion by outlining how the often-proposed Rube-Goldberg-like mechanisms are likely inconsistent with an energy cycle definition of allosteric regulation.

“Allosteric” was first used to mean the effector that bound to “the other site” (*i.e*., a site distinct from the active or orthosteric site). It was used as an adjective to define a type of regulatory response, implying that the focus was on the regulatory outcome. In fact, “allosteric regulation” was introduced (1) before changes in conformational “state” were discussed as a potential molecular mechanism to derive the regulatory outcome (2). Using a protein example, allosteric regulation is the modified function involving one ligand that interacts in the primary functional site that is caused when a second ligand is bound to a distinct site on the protein. The primary functional site is an active site or an orthosteric site. The altered function is ligand binding or catalysis. The second ligand-binding site is often called an allosteric site and the second ligand, an allosteric effector. Restated, allosteric regulation is the through-protein energetic coupling between two protein–ligand interactions (3), and these interactions are described by an allosteric energy cycle, as can be seen in the literature describing the general framework of allosteric investigation (4, 5, 6, 7, 8, 9, 10, 11). Despite this straightforward introduction of what defines allosteric regulation, the nomenclature used within the field to describe the general protein characteristics that contribute to an allosteric mechanism can confuse the uninitiated. In this work, we will first carefully evaluate the existing nomenclature to ensure common use of wording (Table 1). Using that well-defined nomenclature, we will then explore how various systems reported in the literature match the energy cycle definition of allosteric regulation, even if the authors of the primary reports fail to consider the associated energy cycles. Through this exercise, our intent is to gain an appreciation for the breadth of responses/phenomena that can be derived from allosteric regulation responses and gain a greater appreciation for what allosteric regulation is. The reader is encouraged to explore the many examples of protein allosteric regulation cited herein for a richer understanding of the practical applications of allosteric investigation (Table 2). In addition to the list in Table 2, specific protein systems will be referred to in the text occasionally to emphasize the empirical utility of the approach to the subject.

**Table 1:** Glossary^a^

Allosteric coupling for a K-type system	This is the ratio of the affinity of the protein for one ligand in the absence of *versus* the affinity of the protein for that same ligand in the presence of a second protein-bound ligand. Allosteric coupling for a K-type system is determined by following the ligand binding affinity for one ligand over a concentration range (zero to saturating concentrations) of a second ligand. Methodologies for fitting these data trends have been extensively presented and used (7, 8, 56, 57). The magnitude of this ratio can be varied by chemically modifying the allosteric effector, mutating or covalently modifying the protein and/or changing temperature, pH, or other solution conditions
Allosteric effector (allosteric modulators)	Upon binding to a protein, these ligands elicit an allosteric response involving a second protein ligand
Allosteric mechanism (allosteric communication) (allosteric interaction)	Changes that (i) occur within a protein upon binding of one ligand and (ii) result in allosteric responses involving a second ligand. This definition does NOT imply continuous connectivity of networks nor single pathways of interactions (5). Also, binding of a ligand in one binding site does not necessitate a change directly in a second ligand binding site when that ligand is not bound. Instead, some area of the protein must be responsive (*i.e*., energetically coupled) to the binding of both types of ligands (4, 74, 149).
Allosteric site (effector site)	The binding site on the protein to which the allosteric effector binds.
Dynamics	This word reflects the internal protein motions that an equilibrated protein will experience in solution over time and is equivalent to the protein ensemble that is present at any instant of time under the equilibrated condition.
K-type system	A system that demonstrates an allosteric response in which binding of one ligand to a protein modifies the affinity of the protein for a second ligand binding.
Ensemble (protein ensemble)	The full range of in-solution structures is represented under an equilibrium condition. This term implies both the structures present and the statistics of how often each structure is represented to constitute an average. Each liganded state of the protein may have a unique protein ensemble.
Orthosteric site	This a term taken from the receptor field that is a general term for the site of function. In an enzyme, the orthosteric site is the active site. In a receptor, this is the binding site for the agonist ligand.
Reciprocity	The principle that binding of X must impact the ΔG for A binding to E to the same magnitude that the binding of A impacts the ΔG for X binding to E.
A Rube-Goldberg–type mechanism	Temporally sequenced events initiated by effector binding and resulting in a change in the active/orthosteric site)
Structure^b^	Used to describe a single three-dimensional structure of a protein as would be determined by X-ray crystallography. Many structures that are present in solution would constitute the accessible protein ensemble, and a collection of structures would equally define the accessible dynamics of the protein in solution.
V-type system	A system that demonstrates an allosteric response in which binding of an allosteric effector to an enzyme alters the catalysis (*k*_cat_ or *V*_max_) of the enzyme. Although not the focus here, some V-type allosteric mechanisms might be analogous to the K-type allostery involving changes in ligand affinity. Such mechanisms would depend on the catalytic rate-limiting step and/or one of the two relevant binding events involving the transition state. For a V-type system, the allosteric outcome is quantified by comparing the *V*_max_ determined at zero effector *versus* the *V*_max_ determined at saturating concentrations of effector (8).

a Largely taken from Ref. (4) but with modifications and additions.

b This definition for structure is more restrictive than that used in Ref. (4) and equivalent to that previously used for conformational substate.

**Table 2:** Representative proteins referenced herein for useful insights into properties of homotropic cooperativity and heterotropic allosteric regulation^a^

Protein	K-type	V-type	Reference
Arsenic repressor	X		(12)
Metalloregulatory "metal sensor"	X		(13)
Phosphofructokinase	X		(14, 15, 16, 17, 18, 19, 20, 22, 23, 24, 25, 67, 74, 100, 139, 140, 185)
Carbamoyl phosphate synthetase	X		(21, 26)
Calmodulin	X		(27, 28, 29)
Phenylalanine hydroxylase	X		(30)
Tyrosine hydroxylase	X		(31)
Biotin repressor	X		(32, 33)
Pyruvate carboxylase	X		(34)
Hemoglobin	X		(3, 4, 5, 6, 7, 8, 9, 10, 11, 12, 13, 14, 15, 16, 17, 18, 19, 20, 21, 22, 23, 24, 25, 26, 27, 28, 29, 30, 31, 32, 33, 34, 35, 36, 37, 38, 39, 40, 68, 69, 82, 107, 108, 119, 120, 121, 122, 123, 124, 125, 126, 127, 128, 129, 130, 131, 132, 133, 134, 144, 145)
Alpha-isopropylmalate synthase		X	(41, 42, 43)
Glycerol kinase		X	(44, 45, 53)
Imidazole glycerol phosphate synthase		X	(46, 47, 48, 49, 50, 51, 52, 54)
Human glucocorticoid receptor	X		(63)
Pyruvate kinase	X		(56, 57, 73, 75, 111, 118, 136, 137, 138, 141, 146, 147, 165, 166, 167, 168, 169, 181, 186, 188, 190)
Nuclear receptors	X		(64)
Lysozyme	X		(66)

a Proteins and protein families listed as well as allosteric mechanism. Please note that neither all proteins are listed in the table nor are all allosteric systems listed, given that nucleotide-based allosteric systems are also within the scope of this review.

## K-type allostery: an energy cycle involving two ligand-binding events

**Figure 1:**
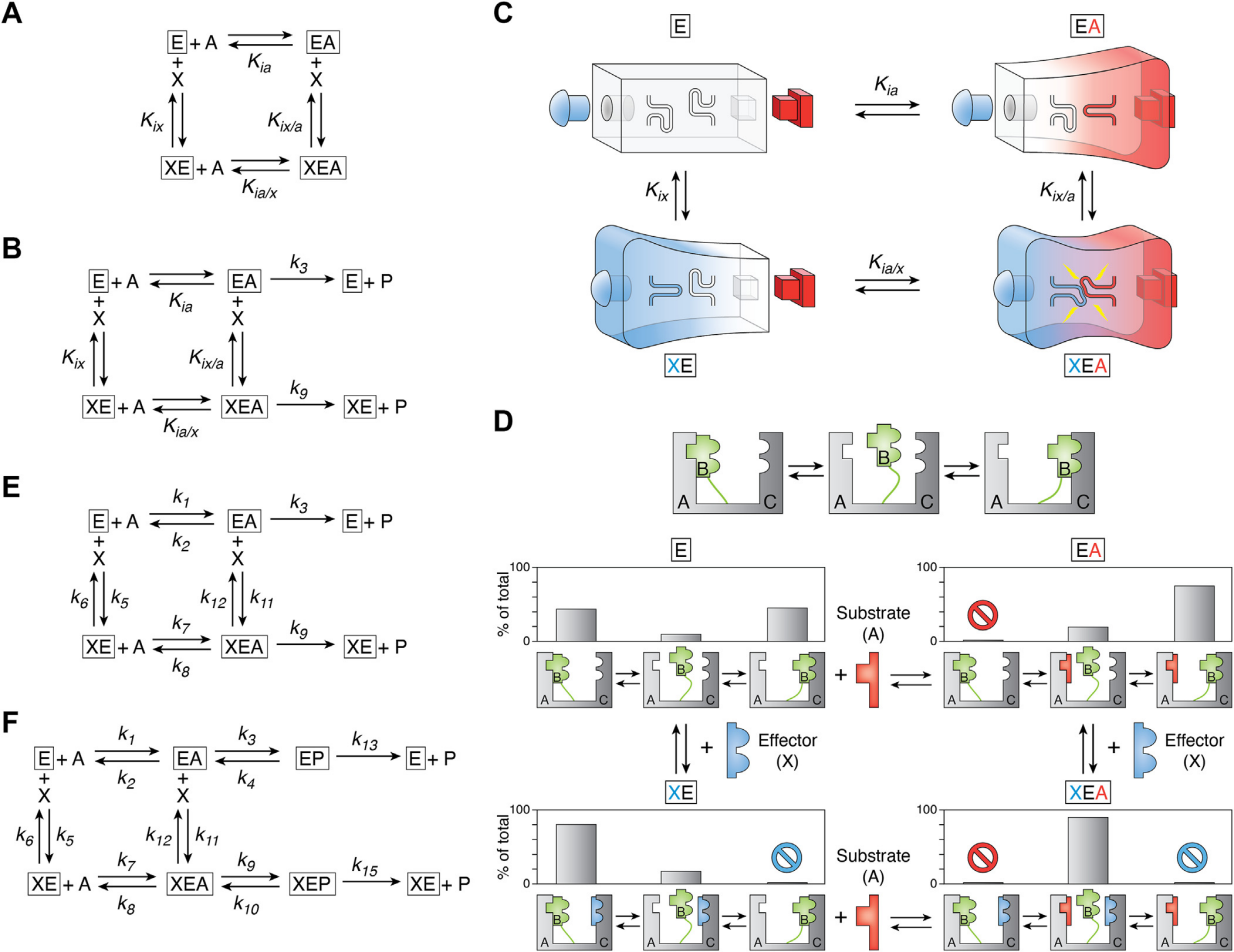
**Allosteric thermodynamic energy cycle.***A*, a simple allosteric energy cycle. To be consistent with the discussions of enzymes in other panels, we will use nomenclatures consistent with enzymes. E will be used for protein. A is substrate and X is effector. *K*_*ia*_ and *K*_*ix*_ are equilibrium binding constants for A and X in the absence of the other ligand. *K*_*ia/x*_ and *K*_*ix/a*_ are binding constants for A and X with saturating X and A, respectively. Note that, because of energy conservation, *K*_*ia*_*K*_*ix/a*_*= K*_*ix*_*K*_*ia/x*_. *B*, allosteric thermodynamic energy cycle for an enzyme. A V-type response indicates that the presence of the allosteric effector (X) influences the rate in which the EA complex is converted to P and released from the enzyme. *C*, an illustration to represent protein changes in various complexes of the allosteric energy cycle. Changes in the protein (*blue and perimeter shape*) result from binding of the allosteric effector (X) (compare E with EX). However, it is possible that most of these changes contribute only to effector binding. In contrast, only a subset of the changes elicited by effector binding may contribute to allostery (shift in *internal blue loop*). Similarly, upon binding of the substrate (compare E to AE), many changes in the protein are caused by substrate (*A*) binding (*red and perimeter shape*), but it is likely that a more limited subset contribute to the allosteric communication (shift in *internal red loop*). Therefore, allostery is only realized when the effector and substrate bind simultaneously in the XEA complex (*red and blue loops*). Importantly, changes in this protein cartoon represent changes in the average conformation and/or dynamics of the protein in solution. This panel has been recreated from Ref. (149) and is intended to convey the same information as a figure in Ref. (4). Importantly, the binding of X does not necessarily result in changes in the binding site of A but instead can alter some “overlapping area of influence” that is also energetically coupled to the A binding event. *D*, the allosteric energy cycle with each enzyme complex to be an ensemble of structures. This panel has been recreated from Ref. (5), where it is discussed in more detail. For this illustration, each complex is allowed to have three structures in the ensemble (see enlarged view above the energy cycle), and the same structures are represented in each complex in the energy cycle. Within the energy cycle, the statistical sampling of structures in the ensemble (*i.e*., % of total) is shown as a bar graph at the top of each enzyme complex representation. The respective structures themselves are below that bar graph. The hypothetical protein has two stationary domains marked as A and C and a domain B that is attached by a flexible tether. Domain–domain interactions and protein–ligand interactions in this simplified model are restricted to shape complementation. Ligands bind competitively with the docking of domain B to the other two domains. Once one docking option is removed *via* ligand binding, the B domain will equilibrate in the new lowest energy interaction. The conetics for the transition can be unique in each individual protein molecule. By considering the unique statistical sampling of all four enzyme complexes, it can be seen that the average conformation is unique among those complexes. This oversimplified illustration is intended to initiate thoughts about how the ensemble might change in an allosteric mechanism; mechanisms other than competitive domain-ligand bind and more complex ensembles are anticipated (5). *E*, allosteric thermodynamic energy cycle, recast in microscopic rates. *F*, allosteric thermodynamic energy cycle, recast in microscopic rates and with catalysis and product release rates explicitly considered.

Allosteric responses can be divided into subclasses based on several criteria. A first example is subdivision based on the regulatory outcome. A K-type system is one in which the binding of an allosteric ligand results in an altered affinity (*K*_app_) of the macromolecule for a ligand that binds in the active/orthosteric site. Therefore, a K-type allosteric system is defined as how a protein (E for enzyme) binds the first ligand (A) differently when the second ligand (X) is absent *versus* is present (4) (Fig. 1*A*). Herein, we will use A for substrate and X for the allosteric effector. The energy cycle in Figure 1*A* accurately describes allosteric regulation in a number of systems, such as calmodulin, hemoglobin, and phosphofructokinase, among others (Table 2) (12, 13, 14, 15, 16, 17, 18, 19, 20, 21, 22, 23, 24, 25, 26, 27, 28, 29, 30, 31, 32, 33, 34, 35, 36, 37, 38, 39, 40). An energy cycle definition of allosteric regulation is also often referenced as linked function.

Allosteric regulation, as defined in Figure 1*A*, is quantified as a ratio (6, 7, 8, 10, 11):

(1) *Q*_*ax*_ = *K*_*ia*_*/K*_*ia/x*_ = *K*_*ix*_*/K*_*ix/a*_,

Where *K*_*ia*_, *K*_*ia/x*_, *K*_*ix*_, and *K*_*ix/a*_ are defined in Figure 1*A*. *Q*_*ax*_ quantifies the extent of the allosteric response, that is, the magnitude of how much X binding influences the binding of A or how much A binding changes when X is absent *versus* is present. Because *Q*_*ax*_ is a ratio of equilibrium constants (and indeed is an equilibrium constant itself for the disproportionation equilibrium (8)), it can be converted to *ΔG*_*ax*_, constituted by both *ΔH*_*ax*_ and *TΔS*_*ax*_ terms.

## V-type allostery: an energy cycle involving two ligand-binding events with enzymatic turnover

Rather than altered ligand binding, a second allosteric outcome is altered catalytic function in an enzyme: altered *V*_max_. For V-type systems, the catalytic conversion of substrate (A) to product (P) is unique between EA and XEA complexes (Fig. 1*B*; see *k*_3_ and *k*_9_). The allosteric function is then quantified as

(2 *W*_*ax*_ = *k*_9_*/k*_3_)(8)

where *k*_3_ and *k*_9_ are as defined in Figure 1*B* (7, 8). Several V-type systems have been characterized using an allosteric energy cycle that includes changes in the rate of catalysis (41, 42, 43, 44, 45, 46, 47, 48, 49, 50, 51, 52, 53, 54). A purely V-type response would be observed if *Q*_*ax*_ *=* 1 and *W*_*ax*_ *≠* 1. In these enzymes, binding of the effector to the protein shifts the dynamics in the equilibrated protein to favor proceeding through the rate-limiting step at a rate of *k*_9_ instead of at the rate of *k*_3_, effectively altering the apparent *k*_cat_. In contrast, a purely K-type response would only shift the substrate affinity (or apparent substrate affinity; *K*_*ia*_ to *K*_*ia/x*_). Interestingly, enzymes can show simultaneous V-type and K-type responses to one effector or show either a pure V-type response or a pure K-type response.

As explored later, V-type responses can be a result of altered product release. However, it is also possible that a V-type response is due to different probabilities that a transition state complex is formed in the EA *versus* in the XEA complex.

## Simulated data to K-type and V-type allosteric responses

**Figure 2:**
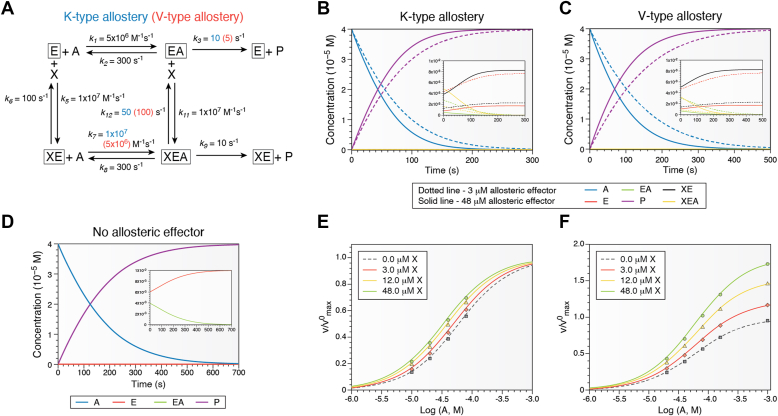
**Simulations of K- and V-type allostery.***A*, rate constants used for simulations, with K-type rate constants shown in *blue* and V-type rate constants shown in *red*. Rate constants that were consistent are shown as *black*. In these schemes, *Q*_*ax*_ = 2 and *W*_*ax*_ = 2. *B*–*D*, populations of each species from *A* as a function of time for K-type (*B*) and V-type (*C*) allostery, shown for two different concentrations of effector X. *D*, the populations for no effector are also shown. The main graph in each panel shows the consumption of substrate and formation of product as continuous kinetic traces, whereas the *inset* shows the concentrations of enzyme species. The starting enzyme and substrate concentrations were 0.1and 40 μM, respectively. *E* and *F*, initial rates from a series of simulations are plotted relative to the maximum rate with no allosteric effector ($v/v_{\max}^{0}$) for K-type (*E*) and V-type (*F*) allostery. Each color corresponds to a different concentration of allosteric effector (X = 0–48 μM). The curves are drawn assuming a steady state concentration of EA and XEA, with fast equilibrium occurring within the thermodynamic cycle. Simulated kinetic data were generated using Kinetiscope (https://hinsberg.net/kinetiscope/).

Figure 2 includes simulated data for both K-type and V-type allosteric regulation of an enzymatic response. In these examples, *Q*_*ax*_ = 2 and *W*_*ax*_ = 2, respectively. Reasonable values for each rate constant are used, and the schemes are kept as similar as possible (Fig. 2*A*). For a system with 0.1 μM enzyme and 40 μM substrate, kinetic simulations can be performed for various concentrations of the allosteric effector X. Here, we used Kinetiscope software (available free of charge at https://hinsberg.net/kinetiscope/) to calculate the populations of all species for K-type, V-type, and no allostery for this system (Fig. 2, *B* and *C*) for continuous kinetic response. The allosteric regulation for both these synthetic systems is modest; nevertheless, a significant change in the initial rate is observed for both K- and V-type allosteric schemes relative to the nonallosteric system (Fig. 2*D*). In both systems, the populations of EA, XE, and XEA are also similar as shown in the inset. However, the behavior over a range of substrate and effector concentrations is very different, as demonstrated by initial rate data simulations (Fig. 2, *E* and *F*). An important note for K-type responses is that all activity responses converge at high substrate concentration, indicating that allosteric outcomes cannot be observed at these concentrations.

## The nomenclature relatedness of “allosteric” and “cooperative”

The terms “allosteric” and “cooperative” have been used in various ways throughout the literature, sometimes interchangeably and sometimes with different meanings. When used interchangeably, some authors have added homotropic and heterotropic to distinguish between the relationship of the chemical natures of the two ligands. To maximize contrast in meaning, we use “homotropic cooperativity” to reference a system where X and A are chemically identical, and both bind to equivalent sites on two different subunits of a multimer. Homotropic cooperative systems in which two identical ligands bind to two unique sites on the protein are also known (55). In experimental studies of positive homotropic cooperative systems, because of the chemical identity of the two relevant ligands, the two ligands are added simultaneously. The first site to be occupied causes a change in the protein that results in the second ligand binding with higher affinity and/or being associated with improved catalytic turnover, to result in an observed sigmoidal response. For an enzyme, the sigmoidal response considered in isolation does not offer insights into whether the homotropic cooperativity is an outcome of a V-type system or a K-type system, since both can generate identical sigmoidal responses. In contrast to homotropic cooperativity, “heterotropic allosteric regulation” describes a system when X and A are chemically distinct and bind to unique sites on the protein. In the experimental study of these systems, the concentrations of A and X can be independently controlled as means of determining how each binds to the protein both in the absence and in the presence of the other ligand (*e.g.*, determine A binding to E when X is present) (56, 57). Although we acknowledge that mathematically homotropic cooperativity and heterotropic allosteric regulation can be considered the same, the experimental designs to study these two cases and the observed outcomes are distinct. Most examples referenced herein (both K-type and V-type) are for heterotropic allosteric systems.

## What to expect as an allosteric mechanism, using well-defined terminology

The energy cycle representation of K-type allosteric regulation focuses on the unique properties that exist among the four protein complexes represented in the cycle (Fig. 1*A*). For our discussion, we will use “dynamics” to refer to the wiggles and jiggles—motions that constitute the Boltzmann distribution—that any one equilibrated protein complex experiences. (In simple terms, a Boltzmann distribution is a description of the probability that each structure in the ensemble will be occupied at a given temperature.) Within an equilibrated solution, the dynamics that each individual protein will explore over time is equivalent to the ensemble of structures that are represented by all protein molecules present at one snapshot of time. The average of all structures that are represented in that ensemble constitutes the protein “conformation.” When ligands bind, we anticipate that the protein conformation (Fig. 1*C*) (4) and/or the equilibrated dynamics will be modified (Fig. 1*D*) (5). Most often, changes in dynamics are expected to be associated with a change in the average conformation. (The exception is when sampling rates and magnitudes of the amplitudes of change are directly opposed so that changes in dynamics do not alter the average conformation (58).) As examples, when comparing the free enzyme E to the EA complex in Figure 1*C*, changes in the average conformation may arise because of changes in positions of side chains and secondary structures, in internal protein motions, in localized folding and unfolding events (*e.g*., biotin protein ligase) (59, 60, 61, 62, 63, 64, 65), in internal protein cavities (*e.g*., phosphofructokinase) (66, 67) and in ordered waters (*e.g*., hemoglobin) (68, 69, 70). However, rather than equating allosteric regulation with ligand binding (which is the comparison of only two enzyme complexes as exemplified in the E and EA comparison), the energy cycle approach defines allosteric regulation of a K-type system as a change in the binding of A caused by the presence of bound X. Therefore, as represented in Figure 1, *C* and *D*, those changes in conformation and/or dynamics that are observed when A binds to E are expected to be different from those changes in conformation and/or dynamics that are observed when A binds to the XE complex (a comparison of XE to XEA). In short, from the energy cycle definition of allosteric regulation, we anticipate that the molecular details that constitute an allosteric mechanism are a difference of a difference among four equilibrated complexes. Thus, the energy cycle definition of an allosteric system does not in itself provide any insights into the allosteric mechanism but provides a framework for interpreting observations from various biophysical techniques. The strength of the energy cycle is that it does not impose restrictions on what conformational changes or dynamic changes might constitute the allosteric mechanism.

In this introduction into what we expect an allosteric mechanism to be, we have provided very exact definitions for our use of conformation, dynamics, and an ensemble. At first glance, it may seem excessive for us to define common terms like “conformation.” However, the importance of this level of precision becomes apparent when considering one particular contrast in the literature. In the early studies from one laboratory, “conformation” was implied to reference the average of the ensemble, which intrinsically includes a consideration of dynamics (71). In that early study, the group discussed how allosteric regulation could be present even without a conformational change (*i.e*., allosteric regulation because of changes in the distribution of protein dynamics without a change in the average of the ensemble, consistent with earlier proposals (58)). In a later work, the same research group implied that “conformation” was equivalent to one structure (*i.e*., as determined by X-ray crystallography) and then argued that function requires a conformational change (72). As emphasis, the first publication implied “conformation” to be the average of a structural ensemble *versus* the second publication implied that a single structure defines “conformation.” The contrasting conclusions from the two publications that appear to result from differences in what is implied by the term “conformation” emphasize the need to use well-defined terms. Importantly, we acknowledge that terms like “conformation” are defined differently in different fields. In solution studies, a signal for a protein solution (*e.g*., a spectroscopic or a small-angle X-ray scattering signal (73)) is representative of a conformation and that signal reflects an ensemble average. On the other hand, the computational field equates conformation with a defined structure in which every atom has a fixed geometric relationship to other atoms. While different terminologies exist in different contributing fields, the collective study of allosteric regulation needs its own standardized language. Again, in our current discussion, we will use “conformation” to refer to the average of the protein ensemble. In this definition, we use “structure” to reference one structural entity in the ensemble. “Structure” was selected for this use because a structure obtained by X-ray crystallography is, at best, only one member in the solution ensemble for that enzyme complex.

As an important note about what an allosteric mechanism is, the energy cycle representation of allosteric regulation places little emphasis on the transitions among the various liganded states of E (5). We previously introduced the word “conetics” (conformational kinetics) to reference the time-dependent transitions among the conformations of the represented complexes (E, XE, EA, and XEA). We introduced this new word to emphasize the contrast between conformational transitions and the equilibrated dynamics (or motions) of any one complex represented in Figure 1 (*e.g*., the dynamics of E). In fact, with regard to a series of events that occur after a ligand is added to a protein solution, every individual protein molecule in a protein solution could use a different “conetic” pathway to achieve the transition from one state of a protein to another state (5). To further appreciate this, consider the oversimplified cartoon model in Figure 1*D*. When one type of ligand binds and eliminates a docking site option for the flexible B domain (because of competitive binding in this example), the B domain then moves to the lowest energy conformation as part of the conetic transition. The trajectory that the B domain follows in this conetic transition may be unique in each protein molecule. Therefore, in contrast to many discussions in the literature, studies of allosteric mechanisms driven by an energy cycle definition of allosteric regulation are NOT primarily concerned with conetic transitions among various liganded states of the enzyme (see later), but instead are, as stated previously, concerned with characterizing the difference of a difference among four equilibrated complexes. Therein lies the need to clearly differentiate between the concept of conetics and changes among the equilibrated dynamics of the complexes: We anticipate that changes among the equilibrated dynamics of the complexes in Figure 1*C* will be very important to understanding the allosteric mechanism.

Several additional points distinguish experimental designs/interpretations associated with the energy cycle definition of allosteric regulation (Fig. 1*C*) as a contrast to what is commonly represented in the literature. First, using the energy cycle to define allosteric regulation does not make any assumption about the conformation of the four enzyme complexes (*i.e.*, there are no restrictions to only two possible “states” among the collection of all complexes). Each complex can be represented as an ensemble of structures, and the conformation and dynamics/ensemble for each complex can be (and likely are) unique. Second, the energy cycle for an allosteric mechanism accommodates, but does not require, a change (dynamics or conformation) of residues that are directly in the A binding site when X binds to E (in the absence of A). Instead, the two binding events must have an overlapping influence on the same area of the protein (Fig. 1*C*) (4, 74), with the active site as only one possible location for that overlapping influence.

In light of these observations, any changes in the conformation or dynamics/ensemble that are identified by comparing only the E and XE complexes are not likely to provide the insights needed to define the allosteric mechanism fully (4). This is due to two reasons (Fig. 1*C*): (1) some of the differences (conformation or dynamic/ensemble) between E and XE are equal to differences between EA and XEA. In this case, the two contributions to the respective binding events will cancel out in Equation 1 because of equal contributions to the numerator and to the denominator. Thus, those binding-associated changes that do not differ when a second ligand is present will not be part of the allosteric mechanism. (2) Some differences will exist only in the EA *versus* XEA conformations (as opposed to E *versus* XE). In this case, comparing E and XE alone would miss contributions to the allosteric mechanism. Therefore, comparisons of only two complexes are not likely to identify all changes that are relevant to the allosteric mechanism (see Ref. (4) for further discussion). In fact, there are very few systems for which equivalent biophysical data types have been generated to allow comparisons among all four complexes (46, 47, 48, 49, 52, 74, 75, 76).

## Allosteric phenomena from the literature

The next goal of this work is to specify characteristics that fall within the framework of the energy cycle–based definition of allosteric regulation. The final two questions in this section require a deeper understanding of the mutually exclusive natures of an energy cycle definition of allosteric regulation *versus* classical two-state models. As such, that needed comparison is inserted immediately before the final two questions.

### Is allosteric regulation limited to proteins? Allosteric DNA, RNA, and allosteric synthetic catalysts

It has been proposed that all dynamic proteins are allosteric (71). Here, we explore how that proposal might relate to nonprotein molecules that exhibit allosteric responses. First, consider that the idea that all dynamic proteins are allosteric is based on an alternative and purely structural definition of allosteric regulation as being equivalent to a ligand-induced change in conformation (71). By that structural definition, a single ligand-binding event that causes altered conformation or altered dynamics on the opposite side of the protein would be included as allosteric (4). This drastically contrasts with our focus on the “other binding site” and systems with two energetically coupled ligand interactions. We are not alone in outlining the contrast; the current recommendation for receptor allostery is that allosteric regulation be “reserved for instances where the properties of one ligand are altered upon binding of a second ligand at a nonoverlapping and topographically distinct site” (3). Importantly, this restriction maintains the focus that allosteric regulation has a regulatory outcome, for example, altered binding of a second ligand. Working specifically within the energy cycle definition for allosteric regulation, it is interesting to ask whether all proteins can indeed have an allosteric response should a second ligand be created or discovered (continuing this speculation is NOT the subject of this work). As an extension, we can ask if all molecules that can experience some level of “change” because of ligand binding can be allosteric and explore examples of allosteric regulation in nonprotein molecules.

There are now multiple examples in which double-stranded DNA is the macromolecule to which two ligands bind. In these examples, the binding of one ligand alters the other’s binding affinity, although there is no direct interaction between the two ligands (77, 78, 79, 80). In these examples, the energetic coupling between two ligands occurs *via* changes in the DNA. More specifically, when a binding event bends double-stranded DNA and the specifics of the bending in that DNA favor or disfavor a second binding event, that DNA appears to satisfy the requirements of an allosteric system. Even when the four potential complexes were not considered in the original literature report, DNAzymes that have altered catalysis in the presence of a transcription factor, as well as other noncatalytic DNA switches, do meet the qualification of two ligands that will be explained by an allosteric energy cycle (*e.g*., heavy metal ion–based DNA switches) (81, 82, 83, 84, 85).

Although RNA shares a general primary structure with DNA, the global folding of RNA is drastically unique from double-stranded DNA. In fact, folded RNA is more similar to protein in that distant primary sequences can reside nearby in the folded structure. Those RNAs that have enzymatic activity are called ribozymes, and those RNAs that exhibit structural rearrangement upon binding a ligand are called riboswitches. Surprisingly, given the proposed roles for ribozymes and riboswitches in metabolic processes in ancient organisms, there is a stark lack of characterized combined ribozymes–riboswitches in cells (86). Nonetheless, synthetic allosteric ribozymes have now been created that bind a ligand to result in altered enzymatic activity (86, 87, 88, 89). Therefore, folded RNA structures can support allosteric regulation. Allosteric ribozymes have the potential to function by either K-type systems or V-type systems.

Given that DNA and RNA can both support changes through their internal structure to give rise to energetic coupling of two ligand-binding events that occur at distant locations, we can ask: do other types of macromolecules/compounds also support allosteric regulation? Again, there are examples that indicate even synthetic chemical systems can support allosteric regulation, such as metal ion tuning of an artificial phosphodiesterase (90, 91, 92, 93). These systems can be summarized as a chemical system that binds two different types of ligands where the presence of one ligand alters the binding affinity for the second ligand.

Collectively, allosteric regulation is not limited to protein systems. The allosteric regulation represented in DNA, RNA, and synthetic chemicals all involve interactions with two ligands, meeting the requirements of the energetic energy cycle description of an allosteric system.

### Is allosteric regulation dependent on the pre-existence of a protein fold? Allosteric regulation in intrinsically disordered proteins

Is allosteric regulation dependent on specific structural elements within the protein (or other macromolecules), or is allosteric regulation simply dependent on more generalized “differences” among the complexes in the energy cycle of Figure 1? We find that the latter must be the answer because there are examples of allosteric regulation in intrinsically disordered proteins and proteins with intrinsically disordered regions, such as human glucocorticoid receptor, among others (59, 61, 94, 95, 96, 97, 98). Importantly, in these examples, it is thought that the disordered proteins fold upon binding to their ligand or protein-binding partner (“partner”). Therefore, a ligand that influences the disordered protein’s folding before binding to a partner or that prevents the ability of the disordered protein to fold in the same way in the presence of the partner defines an allosteric system. In this example, the intrinsically disordered protein binds to both an effector and a protein–protein partner and thus defines a two-ligand binding system that can be described by the energy cycle for a K-type system. Binding-induced unfolding can also play a role in allosteric systems (99). It is conceivable that an effector can also cause an intrinsically disordered protein or protein region to fold into a form that has a higher catalytic activity to result in a V-type response.

### Is a *V*_max_ response the only example of an allosteric modification of a rate? Allosteric changes in rate constants

As noted previously, there are many examples of V-type allosteric systems (*e.g*., imidazole glycerol phosphate synthase, among others) (41, 42, 43, 44, 45, 46, 47, 48, 49, 50, 51, 52, 53, 54). The fact that V-type systems alter the *k*_cat_ rate rather than a binding constant leads us to consider whether allosteric regulation alters other rates in an enzymatic system. To address this, we first recast binding constants as microscopic rate constants (Fig. 1*E*). We can then ask for a K-type system, whether an allosteric regulator modifies the *k*_on_ (*k*_1_ *versus k*_7_) or the *k*_off_ (*k*_2_ *versus k*_8_) (or both). Although we are unaware of extensive studies of both *k*_on_ and *k*_off_ values for the same system, both a K-type allosteric activator and a K-type allosteric inhibitor alter *k*_on_ in a bacterial phosphofructokinase system (100).

Of course, an enzymatic reaction itself can be divided into several microscopic rate steps, each with its own microscopic rate constant. For example, we can expand the Michaelis–Menten representation of the enzyme reaction in Figure 1*E* to separate enzymatic turnover into catalysis and *product* release (Fig. 1*F*). We can then consider whether V-type systems can result from allosteric regulation of the product release (*k*_13_ *versus k*_15_ in Fig. 1*F*). Indeed, there are examples in the literature that identify an allosteric response because of modified product release (*e.g*., alpha-isopropylmalate synthase) (41, 42, 43, 101). It is interesting to draw potential parallels between a V-type system that alters product release with altered substrate affinity in a K-type system.

With the recognition that an allosteric outcome influences microscopic rate constants, many more complex scenarios can be envisioned. There are now examples of two-substrate systems in which one of the two substrates serves as an allosteric effector to modify product release of the second product (102, 103), as well as other examples where energetic coupling may be intrinsic to catalytic mechanisms (*i.e*., one substrate in a distinct fraction of the active site is energetically coupled to function in a second distinct fraction of the active site) (34, 104, 105). Of course, the question remains whether such mechanisms are considered allosteric because of both ligands binding in the same active site. However, we cannot simply dismiss the idea that an allosteric energy cycle involving two unique sites is not part of an enzymatic response: in *Escherichia coli* phosphofructokinase, one type of substrate in one active site can regulate binding of the second substrate in a second active site (18), thus satisfying all aspects of the energy cycle definition of allostery.

Given the examples included in this section, clearly, allosteric effectors can target individual rates.

### Are there upper limits or lower limits to the size of ligands in an allosteric system? Antibody ligands and allosteric photoswitches

An allosteric protein system in which two different antibodies bind to two unique sites on the protein provides an example when both ligands are relatively large (106). For an example of an exceptionally small ligand, we can consider the proton that is at the heart of the long-studied Bohr effect in hemoglobin (107, 108). (The Bohr effect is a pH dependence of oxygen binding by hemoglobin.) However, as yet a smaller ligand, a photon is one of the two ligands in the allosteric energy cycle for a photoswitch or other proteins that undergo light-induced conformational changes (109, 110, 111, 112, 113, 114). Of course, absorption of a photon may be better treated like a covalent modification (see more later) than a reversible small-molecule binding event.

A unique question associated with a large ligand (*e.g*., a protein–protein or a protein–DNA interface) is whether a large ligand that makes multiple contacts across a large binding interface can include a through-protein energetic coupling between those multiple contacts. This idea of energetic coupling in a multivalent interaction (115, 116) can be evaluated by dividing that large ligand into fragments. For example, a DNA ligand might be divided into two short DNA ligands representing different contacts with the protein. This example is useful to identify through-protein energetic coupling examples. However, the allosteric energy cycle definition for allosteric regulation is only satisfied when the ligand is indeed divided into two fragments to allow for two discreet binding events. In an *in vitro* assay where two fragments are treated as separate ligands, energetic coupling between the two ligand-binding events would include a regulatory outcome, and this system is consistent with the energy cycle definition of allosteric regulation. However, when the two native binding partners are both fully intact (*e.g*., a protein binding to one intact large ligand), that binding event does not involve two ligands, and the single binding interaction does not result in a regulatory outcome. For the latter intact large ligand scenario, the through-protein energetic coupling that was demonstrated by the use of ligand fragments does not satisfy the energy cycle definition to represent allosteric regulation. Interestingly, this idea of multivalent interactions might then contribute to the idea of avidity, which is the cumulative influence of many interactions between two binding partners.

Rather than dividing a larger ligand, larger allosteric effectors can be built by tethering small molecules identified from fragment screens (117). However, in those tethered-fragment examples, the allosteric coupling is typically monitored between each fragment and a native ligand that binds to an orthosteric site. This is a different design than our example that fragments a larger ligand to evaluate potential through-protein energetic coupling between the fragments themselves.

Fragmentation has also been used to identify energetic coupling between various parts of one protein. As an example, a peptide of the N terminus of pyruvate kinase was shown to serve as an allosteric activator for substrate affinity (118). This design demonstrated energetic coupling between the N terminus and the active site. When the N terminus peptide included a phosphorylation site, the N terminus had greatly reduced affinity to the remainder of the protein, demonstrating how phosphorylation may alter the interaction of the N terminus. In fact, fragmentation/isolation designs to evaluate how different regions of a protein or a ligand (within the same subunit or among subunits in a multimer) are energetically coupled to each other have been extensively exploited in the study of allosterically regulated systems (29, 118, 119, 120, 121, 122, 123, 124, 125, 126, 127, 128, 129, 130, 131, 132, 133, 134, 135). Each application has required the protein to be fragmented to allow treatment of one fragment as a ligand that can regulate the binding of a second ligand. However, only in the fragment format does the system qualify as an allosteric system (4). Through-protein coupling of a binding event with some other part of the protein that is covalently linked does not meet the criteria for two linked binding events. (Modulation of an intramacromolecule interaction *via* post-translational modification to cause altered function in a distinct active site parallels small-molecule allosteric regulation but lacks the dissociation and binding entropy components of small-molecule ligand energy cycles.)

### Is allosteric regulation restricted to interactions between only two ligands? Systems with multiple allosteric effectors

**Figure 3:**
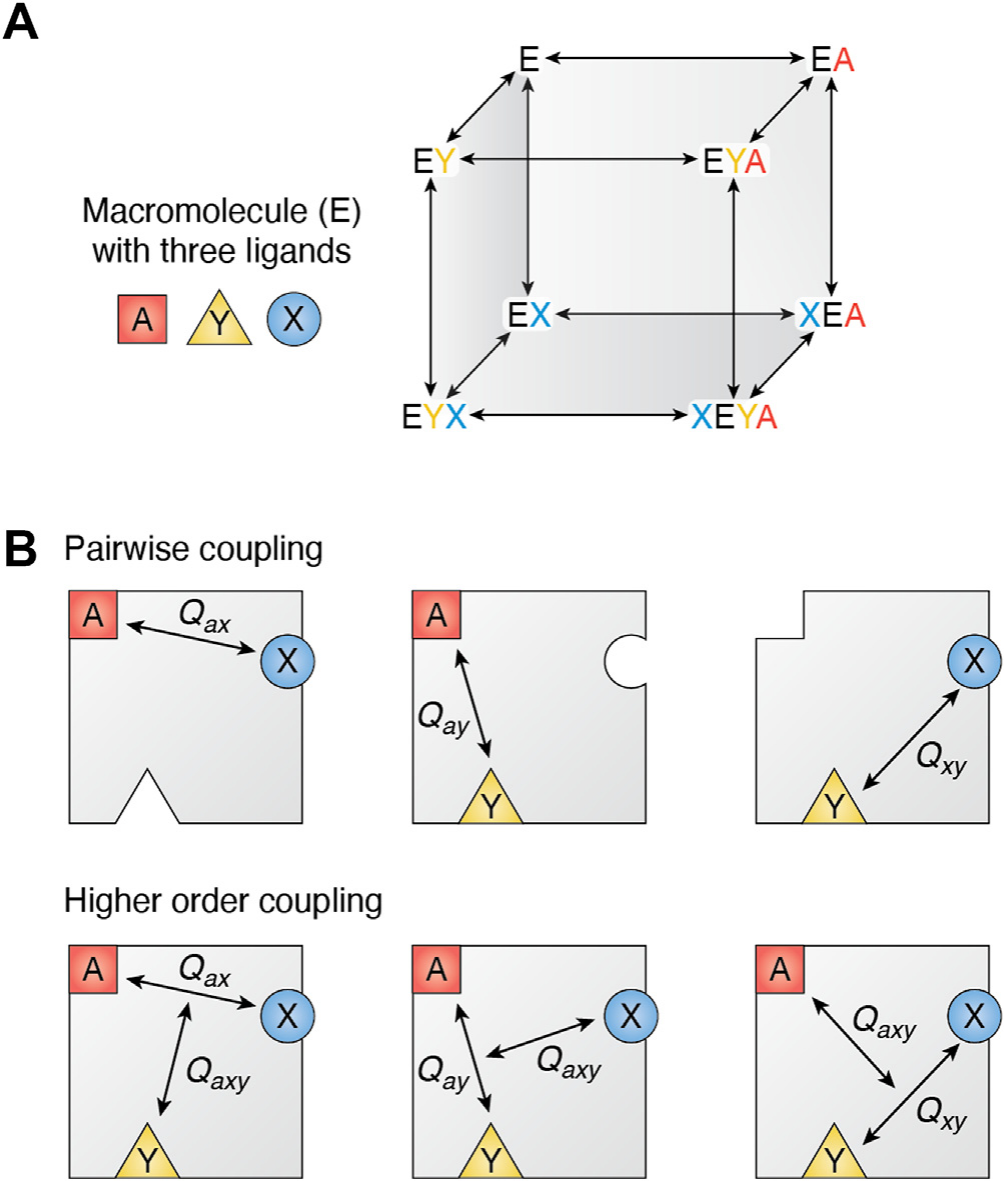
**Allosteric coupling among three ligands that bind to one macromolecule.***A*, a thermodynamic cube representing the binding interaction of a macromolecule (E) with three ligands (A, X, and Y). Each edge line represents a forward and a reverse arrow. *B*, allosteric coupling in a protein (*gray square*) that binds three ligands. *Top*, in a system that binds three ligands, there are three possible pairwise interactions between ligand-binding events. *Bottom*, each ligand can modify the pairwise coupling constant between the other two ligands. In each of the bottom three panels, the higher order coupling constant (*Q*_*axy*_) is equivalent.

In 1963, the novelty of a through-protein energetic coupling of two ligand-binding events provided the impetus for the use of a new label, allosteric regulation (1). It is now well recognized that allosteric proteins often bind many ligands (136, 137, 138). For example, a homotetramer that binds a substrate and two types of allosteric effectors to each subunit binds 12 ligands in total! (17, 57, 108, 139, 140). An array of language is used to indicate the simultaneous influence of more than one allosteric effector (*i.e*., three or more total ligands) (Fig. 3*A*). Logic gate systems include an “and gate”: When two effectors must both be bound to the same protein to activate function involving a third ligand (K-type or V-type) (141). “Synergy,” “negating,” and “mutual effects” have also been used to describe the combined outcomes of multiple allosteric effectors (106, 108, 118, 142, 143).

Within multimeric proteins, hybrid multimers with mutations that reduce the number of ligand-binding events have been useful in evaluating which binding sites regulate which active sites (16, 17, 18, 20, 107, 108, 139, 140, 144). However, a challenge in such systems is the quantification of all coupling constants. Previous studies of heterotropic systems have focused on quantifying the coupling constants between pairs of ligand-binding events (16, 17, 19, 20, 139, 140). In fact, the sum of allosteric coupling quantified in all pairwise interactions is nearly equivalent to the allosteric coupling quantified in the wildtype system. What those previous studies did not extensively explore was the influence of each additional ligand on the pairwise coupling constants (Fig. 3*B*). Those more extensive interactions have been further explored in the homotropic cooperative example of hemoglobin (36, 39, 107, 145). One must also keep in mind that when one of three (or more) ligands is at a subsaturating concentration, then subsaturating effects can influence observed pairwise coupling (discussed in more detail elsewhere (6, 136)). Although quantifying all coupling events in a system that binds more than two ligands can be challenging, these types of systems likely have physiological relevance. As a physiological example, in the study of the M_2_ isozyme of pyruvate kinase, the concentration of the allosteric activator fructose-1,6-bisphosphate is likely sufficiently high that the binding site on the protein is always occupied. Thus, any regulation by the second allosteric effector, an amino acid, depends on the combined regulation caused by fructose-1,6-bisophosphate and the amino acid (146, 147, 148).

### Is the change in “binding” used to describe a K-type allosteric regulation defined by a change in the binding affinity or a change in how a ligand coordinates to the protein? Systems with silent coupling

We have relied heavily on change in binding affinity to define an allosteric system. In fact, a change in substrate binding affinity when an effector is added is often used to identify allosteric systems. That leaves us to consider a system for which the addition of an effector makes a substrate coordinate to the protein differently without altering the binding affinity (149, 150, 151, 152, 153, 154, 155, 156, 157, 158, 159, 160, 161, 162). In other words, the binding affinities for substrate binding to the protein are equal whether the effector is or is not present. Restated again as a question: If a system has structural changes as identified in the difference-of-difference comparisons outlined by Figure 1*A*, but *K*_*ia*_ and *K*_*ia/x*_ are equal, is that an allosteric system? We have previously noted a range of terminologies to describe such examples (149). These examples demonstrate how often the word “binding” is used without distinguishing between the coordinating interactions of a protein and a ligand *versus* the binding affinity that results from that coordination. In our own work, we have used “silent coupling” to describe an implicated change in binding coordination but without a change in substrate affinity (149). Such silent coupling is described by the energy cycle represented in Figure 1. Note that because of the contribution of temperature to the *TΔS*_*ax*_ component of *ΔG*_*ax*_, by assaying at different temperatures, an allosteric response may be observed in a system that is originally identified to have a silent coupling response (149). Therefore, the question that titles this section may be a semantic argument, but it does highlight several important caveats in what sets of words best define allosterically regulated systems.

### Is “cooperativity” defined by a sigmoidal response or is it the coupling between two binding events? The glucokinase example

Not all phenomena that have been called “allosteric” can be described by the energy cycles with two simultaneously bound ligands. A sigmoidal response is sometimes referred to as cooperativity without considering the number of ligand interactions that give rise to that response. Glucokinase is a single subunit with a single active site, yet it has a sigmoidal activity response to a concentration range of the substrate (163, 164). The current explanation for how a single subunit/single active site gives rise to a sigmoidal response is based on relative rates between two steps in the catalytic cycle. In the absence of substrate, the enzyme resides in a conformation with low affinity for substrate. Once substrate concentration is sufficiently high, this low-affinity (and/or low enzymatic activity) form binds one substrate molecule in the single substrate-binding site. That binding event causes global changes to the protein, resulting in a form with a significantly higher affinity for the substrate (and/or higher enzymatic activity). Once catalysis has occurred and the product has been released, whether the enzyme reverts to the low substrate affinity form or binds another substrate molecule depends on competing rates. The rate of binding to the next substrate molecule is dependent on the concentration of substrate. Thus, at low concentration, the rate of binding the second substrate molecule is slower than the rate of the enzyme reverting to the low-affinity form. At higher substrate concentration, the high-affinity enzyme form binds a new substrate molecule faster than the rate of returning to the low-affinity form. Therefore, a sigmoidal response is observed over a concentration range of substrate. To emphasize the point, the higher affinity is toward a second substrate molecule that will bind in the same single active site once the first substrate binding/catalysis/product release cycle has been completed. Although this system gives rise to a sigmoidal response similar to the sigmoidal response of a homotropic cooperative example, glucokinase does not meet our criteria for an energy cycle in which two ligands can simultaneously be bound to the macromolecule.

## Contrasting the mutually exclusive natures of an energy cycle definition of allosteric regulation *versus* classic two-state models

Up to this point, we have only minimally alluded to “classical” two-state models of allostery. That minimal use has been for the intent of maintaining the current focus on the two-ligand thermodynamic energy cycle description of an allosteric system. However, in the literature, several concepts have been associated with allosteric regulation based on defining or implying that allosteric mechanisms are represented by classic two-state models (5). Therefore, our discussion of those associated concepts requires a deeper consideration of two-state models. A two-state model only allows for two states, which removes a need for the ensemble-based distinction between conformation and structure. For this discussion (and any reference to two-state models made previously), we use protein “state” to be consistent with the historical description of two-state models.

The original two-state model is commonly referenced as the Monod–Wyman–Changeux model or the concerted model (2). That model was proposed as a “plausible” model to describe the phenomenon of homotropic cooperativity. In that model, the unliganded protein, with more than one equivalent binding site for a single ligand type, has low affinity for the ligand. When the first binding site is filled *via* ligand binding, the protein changes to a form with high affinity for the ligand. The second binding site then binds to a second ligand molecule with increased affinity compared with the first binding event. The two protein forms are denoted as the T state and the R state (E^T^ and E^R^, respectively).

**Figure 4:**
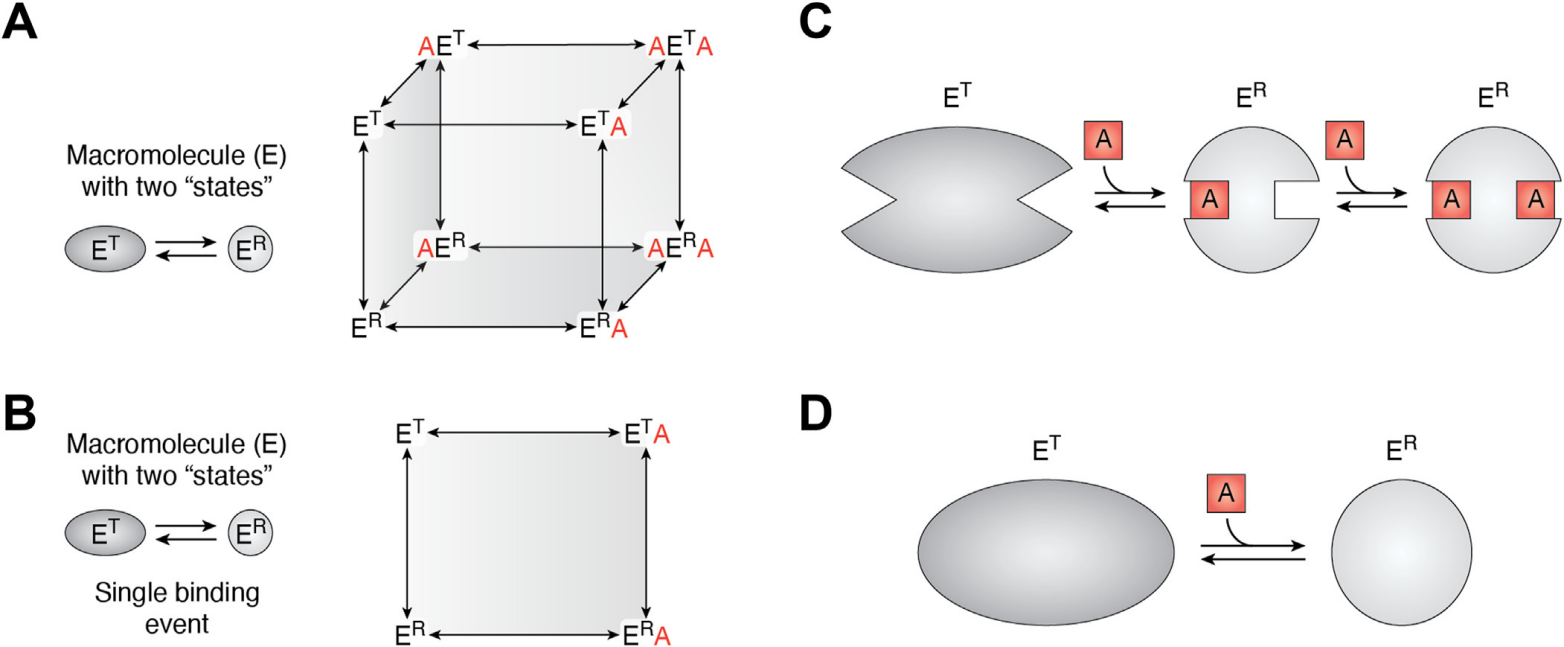
**Four common representations of a two-state model to explain homotropic cooperativity.** In *A* and *B*, each edge line represents a forward and a reverse arrow. Although panels *A* and *B* take on the same geometric shapes as Figures 1*A* and 3, respectively, they represent very different ideas, with a focus on changes in protein states here and a focus on different liganded complexes in the earlier figures. *A*, an energy cube detailing both binding events to the two enzyme “states.” Although this presentation represents more of the two-state model details, the limitation to only two states is more restrictive than the accommodation of the two ligand energy cycles presented in Figure 1*A* that allow each enzyme complex to be an ensemble of structures/states. *B*, an energy cycle that focuses only on a single binding event and the change in the enzyme’s state. This representation does not consider two ligand-binding events and is inconsistent with the energy cycle definition of allosteric regulation. *C*, a sequential binding that recognizes the equivalence (because of structural symmetry of the protein and the chemical identity of the two A molecules) of the two singly liganded enzyme states. In other words, the two complexes in *A* that are labeled as AE^R^ and E^R^A are equivalent and are represented by the single E^R^–A complex in *C*. This representation fails to represent any equilibrium between the E^T^ and E^R^ states within any one enzyme complex. Although this representation does include two ligand-binding events, it fails to represent the energetic coupling between the two ligand-binding events. *D*, a representation of the change in “states,” with a minimal focus on the substrate binding. Several of the faults associated with *B* and *C* also apply to this representation. Color coding in *C* and *D* is used in addition to shape to highlight the different states represented in the models.

The energetic coupling between one ligand type and a change in the “state” of the protein that has been used to discuss homotropic cooperativity is commonly represented in several ways (Fig. 4). Unfortunately, these representations that have all been used in an attempt to express the same model are not equal. The presentations in Figure 4, *B* and *D* both include conformational change but do not consider two separate ligand-binding events. Thus, they do not actually account for homotropic cooperativity between two separate ligand-binding events. Figure 4*C* represents both conformational change and two separate ligand-binding events of the same ligand type. Figure 4*C* also assumes symmetry in the protein and therefore equates AE^R^ and E^R^A that are represented in Figure 4*A*, collectively representing both as a single protein complex. However, as exemplified by the classic hemoglobin model that includes alpha and beta chains in the α_2_β_2_ heterotetramer, symmetry is not intrinsic to all homotropic cooperative systems. In Figure 4*C*, the binding of the second molecule of A is not allowed to exert any additional change on the protein, which seems unlikely. Even the more complete presentation in Figure 4*A* that include the two-ligand energy cycle can be faulted: The top plane in Figure 4*A* that includes four enzyme complexes of E^T^, AE^T^, AE^T^A, and E^T^A is the homotropic cooperativity equivalent to the scheme in Figure 1*A*, where X is replaced with a second molecule of A; this comparison would ignore the T superscript and instead allow each complex in the top plane of Figure 4*A* to be an ensemble. Because the two-ligand energy cycle in that upper plane of Figure 4*A* already describes a homotropic cooperative response, the explicit expansion to an energetic cube, which explicitly defines two states rather than allowing each complex to be an ensemble, makes the model unnecessarily more rather than less complicated (4). In contrast, by leaving the conformation and dynamic features of each of four corners undefined as ensembles (*i.e*., as is true in Fig. 1*A*), the simpler reaction scheme for an energy cycle definition of allosteric regulation accommodates a broader range of changes in the protein than does a two-state model (Fig. 4*A*). The two-state models restrict all accessible protein changes to two states. The representation in Figure 4*A* also introduces an often-considered question of whether a change in the protein “state” happens first or a binding event happens first (*i.e.*, a conetics/transition argument between induced fit *versus* conformational selection), a discussion that is less relevant to an equilibrium mechanism (5). Finally, when applying two-state models to enzymes, a tendency to overlook that the two states can have differences in substrate binding and/or catalytic rates and that either outcome can individually give rise to sigmoidal activity response curves results in a blurred discussion of the underlying change that gives rise to homotropic cooperativity. Despite these criticisms, two-state models have greatly influenced what concepts are often considered as being related to homotropic cooperativity (and to allosteric regulation (5)), and the various representations in Figure 4 are the basis for those ideas.

**Figure 5:**
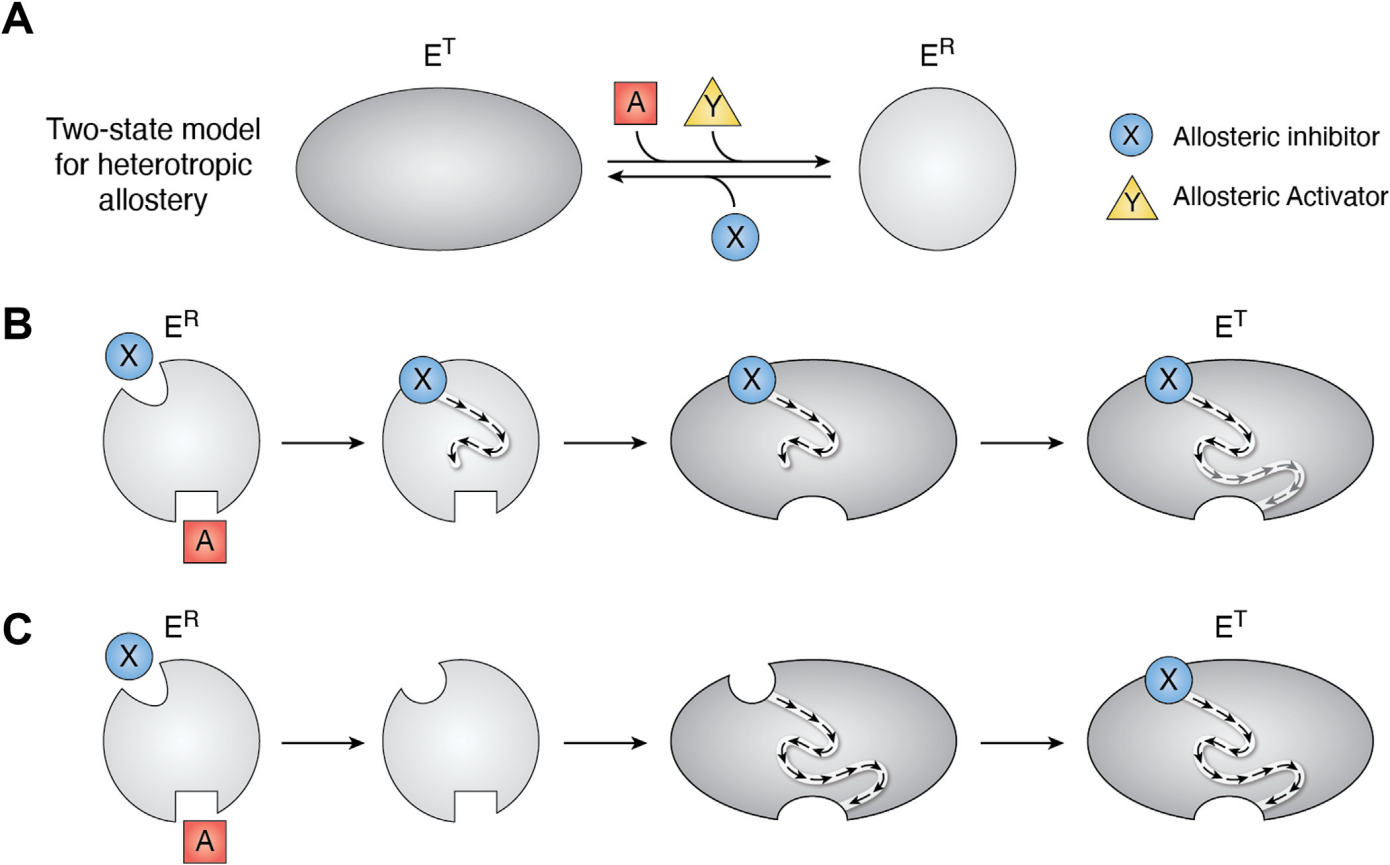
**A simple two-state model for heterotropic allostery.***A*, an extrapolation of the two-state model to include heterotropic allosteric regulation. Here, X is used to represent an allosteric inhibitor and Y is used to indicate an allosteric activator. Equivalent phenotypic responses can be observed for K-type or V-type responses from this model. *B*, an illustration of a Rube-Goldberg–type mechanism for an induced fit transition between the E^R^ to E^T^ transition. The series of changes are represented by *arrows*, and the overall structural change is represented as a single step, followed by additional sequential changes within the protein (*gray arrows*). Although the A-ligand can bind to the E^R^ “state,” it cannot bind to the E^T^ state. *C*, an illustration of a Rube-Goldberg–type mechanism for a conformational selection mechanism of an E^R^ to E^T^ transition. Although induced fit and/or conformational selection may play roles in the forward or reverse transitions between any two of the four complexes represented in Figure 1*A*, a detailed characterization of the order of events in only one of the eight transitions in Figure 1*A* is not likely to provide detailed insights about the allosteric mechanism. Therefore, the focus on how complexes transition (and any focus on induced fit or conformational selection) is minimized by the use of energy cycle definitions of allosteric regulation (5). As an improved focus, the illustrated mechanism in Figure 1, *C* and *D* places the emphasis on comparing a difference of a difference to identify how the binding of one ligand is altered when a second ligand is bound to the macromolecule.

**Figure 6:**
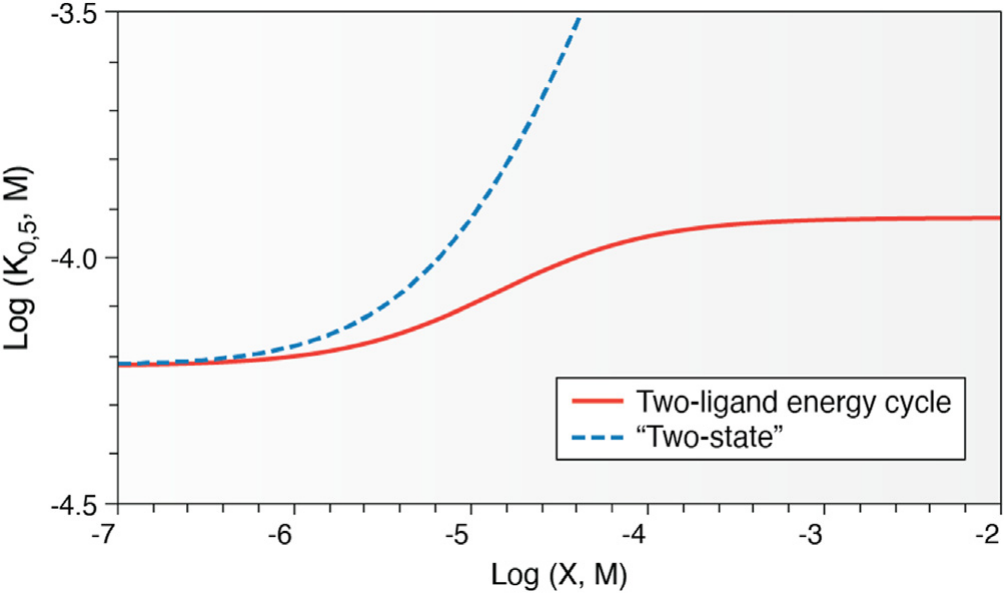
**Contrasting data outcomes from an energy cycle description of allosteric regulation (**Fig. 1***A*) and a two-state model (**Fig. 5**).** This type of comparison has previously been presented (22). This presentation of data includes the binding affinity of one ligand (*e.g.*, a substrate *K*_0.5_) to an allosteric protein over a concentration of the second ligand (*e.g*., an allosteric effector, X). Formation of the upper plateau at high concentrations of X is indicative of the formation of an XEA complex as expected in an energy cycle description of allosteric regulation. However, the absence of an upper plateau does not distinguish between the two descriptions of allosteric regulation: topics like effector solubility limits can prevent observing formation of the XEA complex.

A simple two-state model as developed for homotropic cooperativity is often extrapolated as a generalized model for heterotropic allostery (Fig. 5*A*). At first, such simplistic models seem to provide an easily accessible introduction to a potential allosteric mechanism. However, on deeper evaluation, this representation of a two-state model introduces a range of internally conflicting ideas, some of which are commonly considered and others that are not. (1) If a protein can respond to both allosteric activation and allosteric inhibition, then what is the “state” of the unliganded macromolecule? If only two states are allowed, then the unliganded protein must be some pre-existing mixture of the E^T^ and E^R^ forms (165). (2) If the presence of an allosteric activator has already transitioned all the protein molecules into an E^R^ state (*i.e.*, saturation of the allosteric activator binding sites), then how can homotropic cooperativity for the binding of A remain? In other words, how can a heterotropic allosteric activator also elicit an increase in the homotropic cooperativity associated with substrate binding? (3) If the allosteric inhibitor, X, and the substrate A can only exclusively bind to different states, then how can studies identify the simultaneous binding of X and A to the same protein molecule? (Beyond structures that show simultaneous ligand binding, when the binding affinity of one ligand to the protein is followed over a concentration range of the second ligand, a plateau at concentration of the second ligand is evidence for simultaneous binding of both ligands, see (4) and Figure 6) (4) If a change in the protein’s state is the root of an allosteric mechanism, and if effector binding and that change in state are directly coupled (implying all-or-none allostery), then how can two effectors that bind to the same effector binding site have different binding affinities and elicit different degrees of change in the substrate affinity (73, 166, 167, 168)? (5) Again, because the model in Figure 5*A* seems to imply that allosteric regulation is all or none, then how can mutations to the protein modify the magnitude of the change by which the effector alters substrate binding (169, 170, 171, 172, 173)? (6) How can the allosteric regulation found in one subunit of a homomultimer (isolated by making hybrid tetramers) fail to align with either (a) the concerted two-state model (2) expectation that the complete allosteric response should be contained within one subunit or (b) the sequential two-state model (174) expectation that the complete allosteric response should be elicited independently of which subunit the effector binds to (16, 36)? (7) If heterotropic allosteric activation is derived from the same change in the protein state that gives rise to homotropic cooperativity, then why can the two responses be separated (140)? Clearly, a two-state model, which seems a simple means to introduce what an allosteric model might be, fails to explain many allosteric responses. The big takeaway is that a two-state R and T model is too simplistic to account for the myriad allosteric responses observed in both homotropic and heterotropic systems.

Specifically, consider that the model in Figure 5*A* excludes the possibility for simultaneous binding of an allosteric inhibitor with the substrate. In contrast, the two-ligand energy cycle definition of allostery (Fig. 1) is completely dependent on the formation of the tertiary XEA complex (even when that tertiary complex is not observed because of an experimental limitation, for example, ligand solubility limits). This two-state model is therefore *mutually exclusive* with a two-ligand energy cycle definition of allostery. Synthetic data (Fig. 6) based on the reaction schemes in Figures 1*A* and 5 emphasize the mutually exclusive natures of these two views of allostery, including the distinguishing emphasis on the formation of the XEA complex as an upper plateau in the response.

Consistent with the mutually exclusive natures of the model in Figure 5 and the energy cycle definition of allosteric regulation in Figure 1, those two representations provide vastly contrasting expectations for what an allosteric mechanism is. As introduced previously, the energy cycle definition of an allosteric mechanism focuses on the difference of a difference among the four equilibrated complexes included in Figure 1. In Figure 5, the only concepts that can change are the ratio of E^T^ to E^R^ in the unliganded state and the rates of interconversion between E^T^ and E^R^. Therefore, the outcome of the model in Figure 5 is a focus on the transition between E^T^ and E^R^ complexes as the allosteric mechanism. This focus can be questioned at the onset: For a K-type system that results in modified substrate binding, and emphasizing that binding is intrinsically an equilibrium function, why is a proposed allosteric mechanism focused on the conetics associated with transitions between states?

A secondary challenge comes from the common focus on only one transition, from E complex to the XE complex: How can an evaluation of only one conetic transition, without considering the other seven conetic transitions included in Figure 1*A*, successfully provide insights into an allosteric mechanism? It follows that if, indeed, the focus on an equilibrium mechanism represented in Figure 1*A* deemphasizes the need to focus on conetics, then topics like the highly debated “induced fit” *versus* “conformational selection” pathways for conetic transitions are also de-emphasized in studies of allosteric mechanisms (5).

### Are the many Rube-Goldberg–type mechanisms that are often proposed from comparing structures with and without an allosteric effector consistent with what we expect an allosteric mechanism to be?

Despite the inconsistency of focusing on the conetics of a transition between states *versus* the equilibrium nature of altered substrate binding, transitioning between states in allosteric systems is a common focus in the literature. In particular, *in silico* computational approaches work with well-defined geometric structures. Therefore, two-state models are intrinsic to most computational studies of allosteric systems. One type of report that is common from current computational studies is a predicted series of temporally sequenced events initiated by effector binding and resulting in a change in the active/orthosteric site (Fig. 5, *B* and *C*) (109, 175, 176). This assumed sequence has at times been referred to as an allosteric cascade (177, 178, 179, 180). Perhaps that focus may be logical for a two-state model of allosteric regulation that limits the protein to two conformations and places an emphasis on how the protein transitions between those two conformations to directly alter the active site. We term this type of prediction a “Rube-Goldberg–type” mechanism, after the popular American cartoonist in the 1910s to 1950s, who depicted machines that accomplished a simple task through a series of convoluted consecutive steps. A second common outcome from computational studies is a pathway of an energetically connected network that links the effector binding site with the allosteric site (46, 181, 182, 183). These findings are often discussed with an implication that when an effector is bound, some Rube-Goldberg set of changes along the identified network of amino acid residues will result in modified substrate binding. In contrast to those Rube-Goldberg interpretations, the energy cycle definition of allosteric regulation causes a speculation that each of the four complexes in the energy cycle might have unique networks (181) and/or unique coupled motions within the protein (49). Of course, many proposed allosteric mechanisms based on comparing a structure without an effector bound *versus* with an effector bound are also Rube-Goldberg style mechanisms.

Like the focus on conetic transitions that give rise to proposed Rube-Goldberg mechanisms, a temporal series of events that lead from an effector binding site and result in a modification in the active site is a kinetic mechanism. Kinetic mechanisms are not consistent with the equilibrium nature of allosteric regulation. (We note that studies of kinetic mechanism can be very important when applicable.) Rube-Goldberg mechanisms also tend to ignore a need to account for reciprocity, which is the idea that the influence that X has on the binding affinity of the macromolecule for A must be equal to the influence that A has on the binding affinity of the macromolecule for X, an identity represented in Equation 1. Furthermore, comparing only two structures completely overlooks the need to compare the four complexes included in the energy cycles that define allosteric regulation. Consistent with the concept that Rube-Goldberg mechanisms are not expected based on an energy cycle definition of allosteric regulation, proposed Rube-Goldberg mechanisms have not been supported when tested (16, 22, 184, 185). In fact, blind computational predictions of the consequence of mutations in allosteric proteins have also been unsuccessful (186), which may not be surprising given the current dependence of computational approaches in two-state model/Rube-Goldberg concepts. We find it unfortunate that the extensive use of two-state models in the field has led many to expect Rube-Goldberg mechanisms to be at the root of allosteric outcomes (109, 175, 176, 177, 178, 179, 180). Once such Rube-Goldberg mechanisms have been proposed, they are seldom challenged with rigorous experimental designs. Based on theoretical reasons presented here for why we should not anticipate Rube-Goldberg mechanisms, we strongly encourage the field to design experimental approaches that specifically challenge such proposed ideas in a broader range of proteins.

Even for a V-type allosteric mechanism, we anticipate the enzymatic rates to be associated with complexes that have the allosteric effector either absent or bound (*i.e*., both are equilibrated complexes). In other words, the modified *V*_max_ outcome is due to a change in the probability of the enzymatic transition state complex being formed as a result of altered dynamics in the *equilibrated* effector-bound complex. In contrast, an image that is harder to accommodate in the V-type equilibrated system is an effector-induced Rube-Goldberg mechanism that modifies the active site at the exact moment that catalysis occurs. This inconsistent idea would be a low-probability event, and it would require the effector to cycle on and off the protein at the same or a faster rate as *k*_cat_ to cause an influence on more than a single turnover per enzyme molecule!

### Do altered allosteric outcomes in proteins carrying point mutations offer support for proposed allosteric mechanisms?

In our quest to use data reported in the literature to better outline what an allosteric mechanism is, we cannot overlook the use of mutational studies (5). It is now common to use point mutations to probe protein residue positions that are predicted to have roles in a proposed allosteric mechanism. Few such mutational studies explore whether a range of molecular allosteric mechanisms might be proposed for the same system and how one given residue might be used differently in that range of proposed mechanisms. Instead, any change in an allosteric response in a designed mutated protein is often touted as support for a single proposed mechanism. Furthermore, few mutational probing studies include negative controls, that is, mutating residues that are not predicted to participate in the proposed allosteric mechanism to confirm a lack of modified allosteric function. To challenge the standard applications of mutational probing designs, a whole protein alanine scanning mutagenesis study was completed with a focus on quantifying allosteric coupling constants (*Q*_*ax*_) for each mutant protein. For one allosteric system, replacing ∼30% of the nonalanine/nonglycine amino acid residues with an alanine substitution caused a change in the allosteric outcome (172). If that result represents the potential in other proteins, then in a study where only three positions are probed using point mutations (187), at least one of those residues is highly likely to alter the allosteric outcome, independent of a predicted allosteric mechanism. Therefore, caution should be made in interpreting altered allosteric responses caused by point mutations as support for proposed allosteric mechanisms (5).

## Practical recommendations for evaluating allosteric regulation based on an energy cycle

1. In studies that evaluate functional outcomes like altered ligand binding affinity or modified *V*_max_, evaluate the altered function across a large range of effector concentrations. Because of reciprocity, when effector concentration is held constant while evaluating substrate affinity *via* titration, the effector binding affinity is modified. This makes it challenging to use a single effector titration to determine what effector concentration will be saturating at all substrate concentrations and vice versa. An ideal experimental design simultaneously titrates both ligands (*i.e.*, varying the concentrations of each ligand separately) to confirm saturation for both substrate and effector to quantify allosteric outcomes (172).
2. Report allosteric outcomes quantitatively as *Q*_*ax*_ and/or *W*_*ax*_ values. These quantitative values allow for the magnitude of allosteric outcomes to be compared among, for example, different isozymes of one enzyme (8).
3. When gathering biophysical information to study allosteric mechanisms, collect equivalent data types on all four complexes represented in Figure 1. Evaluate data as a difference of a difference (46, 47, 48, 49, 52, 73, 149, 188).
4. When proposing potential allosteric mechanisms based on biophysical data, considerations should be given to whether those mechanisms account for the equilibrium nature and the reciprocity of the allosteric system (173).
5. Because mutations can either remove or introduce new mechanisms of allosteric coupling and can do so by either contributing to enthalpy, entropy, or both, caution should be used when interpreting mutational outcomes as support for allosteric mechanisms. A range of substitutions should be used in mutational studies (169, 170, 171, 189, 190, 191, 192), and controls should evaluate positions that are both part of and not part of the proposed mechanism (5, 166, 172, 173).
6. Enzymatic activity assays (or binding assays for ligand binding to the orthosteric site) can often be a first-approach experimental design for quantifying allosteric effector/drug binding affinities. Within the need to determine allosteric effector/drug binding affinities, the utility of quantifying effector binding affinity *via* an often already in-hand enzymatic assay (or an orthosteric site binding assay) is commonly overlooked. Specifically for enzymes, the experimental design outlined in point 1 of this section that includes determining an enzymatically determined apparent affinity for the substrate across a range of effector concentrations, intrinsically and rigorously quantifies the thermodynamic binding constants for allosteric effectors/drugs (in addition to quantifying *Q*_*ax*_) (7, 8, 9).

## Conclusions

It is time for our field to fully embrace that the early two-state models of allosteric regulation, which were exceptionally useful in introducing the idea that proteins are not static structures, are in fact interfering with more complete characterizations of allosteric mechanisms. This includes a need to outline new computational tools to evaluate *equilibrium* mechanisms of allosteric regulation (193, 194, 195, 196, 197, 198). To facilitate the training and validation of future computational tools, databases of modified proteins with well-characterized allosteric coupling outcomes (189) are needed. At the same time that we are realizing this need for a change in thought throughout the field, a range of newly characterized systems have expanded our understanding of what types of macromolecules can be allosterically regulated and what type of molecules can serve as allosteric ligands. Collectively, studies of the molecular details of allosteric mechanisms are on the cusp of transitioning into a phase of giant leaps in understanding. The two-ligand energy cycle definition for allosteric regulation stands as a conceptual framework ready to facilitate those leaps.

## Conflict of interest

The authors declare that they have no conflicts of interest with the contents of this article.
